# Micronutrients intake and genetic variants associated with premature ovarian insufficiency; MASHAD cohort study

**DOI:** 10.1186/s12905-023-02865-4

**Published:** 2024-02-04

**Authors:** Mohammad Reza Mirinezhad, Maliheh Aghsizadeh, Hamideh Ghazizadeh, Sahar Ghoflchi, Mohammad Zamiri Bidary, Alireza Naghipour, Gordon A. Ferns, Tayebeh Hamzehloei, Alireza Pasdar, Majid Ghayour-Mobarhan

**Affiliations:** 1 Department of Medical Genetics, Faculty of Medicine, Mashhad University of Medical Sciences, Mashhad, Iran; 2 International UNESCO Center for Health-Related Basic Sciences and Human Nutrition, Mashhad University of Medical Sciences, Mashhad, Iran; 3 Student Research Committee, Mashhad University of Medical Sciences, Mashhad, Iran; 4 Brighton & Sussex Medical School, Division of Medical Education, Falmer, Brighton, Sussex BN1 9PH UK; 5 Division of Applied Medicine, Medical School, University of Aberdeen, Foresterhill, Aberdeen AB25 2ZD UK; 6 Metabolic Syndrome Research Center, Mashhad University of Medical Sciences, Mashhad, 99199-91766 Iran

**Keywords:** Genetic haplotypes, Premature ovarian insufficiency, Tetra ARMS PCR and ASO-PCR, Micronutrient

## Abstract

**Background and aim:**

premature ovarian insufficiency (POI) is defined as the menopause before 40 years of age, and its prevalence is reported to be two-fold higher in Iranian women than the average for woman globally. POI is associated with several cardio/cerebrovascular complications as well as an increased overall mortality. Genetic factors, and serum levels of minerals and vitamin D, have been reported to be related to the prevalence of POI. We have investigated the association between some POI -related genotypes with the serum levels of some important micronutrients.

**Methods:**

One hundred and seventeen women with POI and 183 controls without any renal, hepatic, and thyroid abnormalities were recruited as part of the MASHAD study. Demographic and anthropometric features were recorded and blood samples were collected and processed. DNA was extracted from the buffy coat of blood samples from all participants and 8 POI-related single nucleotide polymorphisms (SNPs) were determined using ASO-PCR or Tetra ARMS-PCR. Serum minerals and vitamin D concentrations were measured using routine methods.

**Results:**

In women with POI, serum copper, phosphate, and calcium were significantly different for those with rs244715, rs16991615, and rs4806660 genotypes, respectively. In our control population, significant differences were also found in serum copper concentrations between different genotypes of rs4806660, rs7246479, rs1046089, and rs2303369. After adjusting for all confounding factors, the women with POI carrying TC genotype (rs4806660) had a lower risk to have serum copper levels < 80 (µg/dL) than those carrying a TT genotype. Furthermore, women with POI carrying GG genotype (rs244715) had a 6-fold higher risk to have serum copper levels > 155 than those carrying AA genotype.

**Conclusion:**

The C and G alleles of the rs4806660 and rs244715 polymorphisms respectively are independently associated with serum copper in women with POI. Further studies are necessary to investigate the association of serum copper and other micronutrients in women and other POI -related polymorphisms.

**Supplementary Information:**

The online version contains supplementary material available at 10.1186/s12905-023-02865-4.

## Introduction

Natural menopause is defined as the cessation of menstruation and the menstrual cycle for 12 continuous months during a women’s lifecycle and that happens without any identified psychological or pathological cause [1, 2]. This condition occurs naturally as a result of deficient serum estrogen due to ovarian ageing and follicular depletion at the average age of 48.78 years [3–5]. Premature menopause (PM), also identified as primary ovarian insufficiency (POI), is defined as menopause arising before the age of 40 years, that occurs naturally, or due to previous history of reproductive system surgery [6, 7]. A recently published meta-analysis estimated 3.60% of women experienced POI [3], whilst, an epidemiological study reported 5.94% of Iranian women develop POI [8] which is 65% higher than the worldwide estimate of POI prevalence. This difference in prevalence of POI may be due to the lower age of menarche in Iran compared to the world mean, which is one of the risk factors for POI [9–12]. Also, the age of development of ovarian cancer is considerably lower in Iranian women than in the developed countries, and treatments such as chemotherapy and other therapeutic interventions may lead to POI [13, 14].

Genome-wide association studies (GWAS) have shown that a polymorphism (rs16991615) of the minichromosome maintenance 8 homologous recombination repair factor (MCM8) gene was concluded to play a significant role on the age at which natural menopause occurs, as a result of aminoacid change (Glu to Lys) in the synthetized protein [15]. Moreover, one of the most significant associations was found in the rs1046089 single nucleotide polymorphism (SNP) in Proline Rich Coiled-Coil 2 A (PRRC2A) gene. As this SNP is responsible for modifying human leukocyte antigen in monocytes (HLA-DRB4) and lymphoblastoid cells (HLA-DQA1), it may be a potential proinflammatory component which causes oocyte depletion and subsequently, an earlier menopause [16]. In addition, the study of Weedon et al. reported that the rs4806660 polymorphism, transmembrane (TMEM) gene, is associated with a higher risk of developing an early menopause [17, 18]. Furthermore, there are several more variants and polymorphisms significantly related to an earlier age of the menopause mentioned in the GWAS [19, 20].

Micronutrients are found to have significant impact on the hormones involved in female reproduction [21]. Vitamin D, via its receptors that are present on the cells of ovary and uterus, might be responsible for female reproductive tract disorders, or effects the regulation of anti-Müllerian hormone [22]. A cohort study of registered nurses from across US, found women who consumed higher amounts of vitamin D and calcium from dietary dairy products, had a lower risk of premature ovarian insufficiency or POI [23]. Furthermore, Kebapcilar et al. in a cross-sectional analysis comparing healthy women and women with non-surgical POI, found lower serum vitamin D and zinc and higher copper in the cases [24]. According to these studies, it appears to be necessary to assess the association between POI -related genotypes and micronutrients, that appear to be related to the function of female reproductive organs.

To the best of our knowledge, there have been no studies addressing the question of whether there is any association between serum minerals/vitamin D and genotypes known to be related to PM/POI. Therefore, we aim to investigate this relationship in a sample of Iranian people who participated in Mashhad Stroke and Heart Atherosclerotic Disorder (MASHAD) study.

## Methods

### Study participants

In this cross-sectional study, 117 women with POI and 183 healthy women were recruited as the case and control groups, respectively. Theses participants were enrolled as part of the MASHAD study that is a 10-year cohort study (2010–2020) and includes a total of 9704 people aged 35–65 years [25]. According to the questionnaire obtained from the baseline study, women who: (1) entered the menopause at 40 and < 40 years of age, (2) had 12 consecutive months without menstrual bleeding, or (3) had a serum FSH > 30 IU/L (repeated 3 times at four-week intervals) were considered as having POI [7]. Women > 40 years old, those with a history of diseases, syndromes, surgeries, and taking drugs that may have affected menstruation, were excluded. Vacutainer® tubes were used for blood sample collection. All participants were on 14-hour overnight fast. Blood samples were centrifuged at 4^o^ C in 5000 rpm for 15 min and the serum separated and stored at -80^o^C for future analysis. Measurement of the anthropometric characteristics: weight, height, body mass index (BMI) hip and mid-upper arm circumference, waist-hip and waist-height ratio were performed using standardized methods [25, 26]. All participants had normal kidney, liver, and thyroid functions.

### DNA extraction and quality controls

Whole DNA of all participants’ genome from 200 µl of their buffy coat samples were extracted by DNA extraction kit (Pars Tous Biotechnology, Mashhad, Iran). Qualitative and quantitative quality controls were done using agarose gel electrophoresis (Pars Tous Biotechnology, Mashhad, Iran) and Nano drop 2000 (Thermo Fisher Scientific, USA) in 280 and 260 nm wavelengths, respectively.

### Allele-specific oligonucleotide polymerase chain reaction (ASO-PCR)

We added ASO-PCR in a total 15 µl reaction volume which consisted of the followings: 2 µl genomic DNA, 1.5 µl water, 1 µl of each primer, and 7.5 µl master mix (Pars Tous Biotechnology, Mashhad, Iran). The PCR conditions were as follows: at 95 °C, we carried out one cycle of denaturation for 7 min, followed by 35 cycles consisting of the following: 30 s for denaturation at 95 °C, annealing for 30 s at 60 °C, and 30s of extension at 72 °C. Final extension was performed for 7 min the same as initial denaturation.

### Tetra amplification refractory mutation system PCR (ARMS-PCR)

Tetra ARMS PCR was carried out on the same composition of 15 µl reaction volume and method of performing PCR which were addressed above for ASO-PCR. Primer1 software [27] was used for designing the primers.

### Minerals and vitamin D measurement

Zinc (Women: 70–114 µg/dL, Men: 73–127 µg/dL), copper (Women: 80–155 µg/dL, Men: 70–140 µg/dL), phosphate (2.5–4.5 mg/dL), calcium (Children: 8.8–10.8 mg/dL. Adults: 9-10.5 mg/dL), magnesium (Infants: 1.4-2.0 mEq/L, children: 1.4–1.7 mEq/L, adults: 1.3–2.1 mEq/L), vitamin D (adults: 10–30 ng/ml) were measured in the blood serum of all study participants. Flame atomic absorption spectrometry (Perkin Elmer model 3030, USA, 1980) was used to measure serum levels of copper and zinc, as previously described [28]. Using Pars Azmun kits (Pars Azmun, Tehran, Iran) and a BT3000 auto-analyzer along with photometric methods, we measured serum calcium and phosphate [29]. According to intensity of purple color radiated originally from calcium and cresolphthalein complexone interaction. We measured serum phosphate by accessing the intensity of color produced by chemical reaction of phosphate, ammonium molybdate, and sulfuric acid [29]. Xylidyl blue photometric method, BT3000 autoanalyzer (Biotechnica, Rome, Italy), and Pars Azmun kits (Pars Azmun, Tehran, Iran) were applied for measurement of magnesium. An enzyme linked immunoassay (ELISA) method was applied to measure 25-hydroxy vitamin D [30, 31].

### Ethics

All participants were made aware of study protocol and signed an informed consent form. Mashhad University of Medical Sciences (MUMS) ethics committee approved the methodology of this project.

### Statistical analysis

Values in this study are shown as frequencies along with the percentage or mean and standard deviation. Chi-square test was used for comparing SNPs’ genotype frequencies between POI cases and healthy controls. Kolmogorov-Smirnoff test used for assessment of normal distribution in the quantitative data. Two-independent sample t-test, one-way analysis of variance (ANOVA) analyses and Tukey tests were performed to compare quantity of normally distributed values between the two subgroups. Moreover, we employed multivariate logistic regression in order not to allow confounders, impact our results. All tests were performed by Statistical Package for Social Sciences software, known as SPSS (IBM Corp. Released 2016. IBM SPSS Statistics for Windows, Version 24.0. Armonk, NY: IBM Corp.), and *P* < 0.05 was treated as significant.

## Results

The participants’ demographic data are summarized in Table 1. This study the mean age of the participants was 54 years. Moreover, details on POI-related polymorphisms and genes that were investigated in our study are shown in Table 2.

**Table 1:** Demographic features and characteristics of the study population

Characteristics	POI cases, N = 117	Control, N = 183	*P*-value
\	\	\	**\**
Waist circumference (cm)	96.96 ± 12.19	93.70 ± 10.44	0.020
Weight (Kg)	67.11 ± 12.03	70.96 ± 10.80	**< 0.001**
Hip circumference (cm)	104.71 ± 9.85	105.57 ± 8.78	0.430
Waist-height ratio	63.55 ± 8.41	60.30 ± 6.84	**< 0.001**
Waist-hip ratio	0.93 ± 0.08	0.89 ± 0.07	**< 0.001**
Mid-upper arm circumference (cm)	30.69 ± 4.47	31.02 ± 4.61	0.550
BMI (Kg/m^2^)	28.78 ± 5.06	29.34 ± 4.22	0.300

All variables had normal distribution and their data are indicated as Mean ± SD (Standard Deviation); POI: premature ovarian insufficiency; SD: Standard deviation; Two-independent sample t-test was done

**Table 2:** Characteristics of polymorphisms associated with POI in which were included in this study

SNP	Gene	Alleles	Chromosome	MAF (1000Genomes)	Method genotyping	Ref
rs4806660	TMEM	T > C	19:55313266	C = 0.330	ASO	[32–34]
rs451417	MCM8	C > A	20:5961353	A = 0.280	ASO	[35]
rs16991615	MCM8	G > A	20:5967581	A = 0.026	ASO	[36, 37, 32]
rs7246479	BRSK1	T > A	19:55312964	T = 0.410	ASO	[15, 38, 39]
rs244715	ZNF346	A > G	5:177076562	A = 0.380	ASO	[39, 34]
rs1046089	PRRC2A	G > A	6:31635190	A = 0.390	ASO	[40–42]
rs2303369	FNDC4	C > T	2:27492549	T = 0.350	ASO	[43, 44]
rs10183486	TLK1	C > T	2:171134461	T = 0.310	Tetra ARMS	[43, 45]

POI: premature ovarian insufficiency; SNP: Single nucleotide polymorphism

### Association of polymorphisms with POI

Of the study population, 117 were confirmed to have a POI. We identified the association between the occurrence of the eight variants and POI in patients and control groups, also the Hardy-Weinberg equilibrium calculated for each SNP. It showed that society is in Hardy-Weinberg equilibrium, rs4806660, rs451417, rs16991615, rs7246479, rs244715, rs1046089, rs2303369, and rs10183486 with a *P*-value of 0.2, 0.1, 0.2, 0.2, 0.3, 0.1, 0.1, and 0.2, respectively (Table 3). As shown from this table, 8 SNPs were selected for this study: rs16991615 (*p* = 0.002), rs244715 (*p* = 0.006), rs451417 (*p* = 0.001), rs7246479 (*p* = 0.002), rs4806660 (*p* = 0.02), and rs10183486 (*p* = 0.02) and were significantly associated with POI. Also, the distribution of genotypes is listed in Table 3. As an example, TT, TC, CC genotypes frequencies of TMEM variants rs4806660 were estimated 34.7% and 65.3%, 39.3% and 60.7%, 66.7% and 33.3% for the POI and control group, respectively. The POI group showed the following BR Serine/Threonine Kinase 1 (BRSK1) gene rs7246479 genotypic distribution; 28% TT, 43% TA and 63% AA, which was significantly different from the controls (71%, 58% and 36% respectively) (*p* = 0.002). Analysis of two polymorphisms at the MCM8 gene locus showed that the rs16991615 and rs451417 variants were similar in frequency for the AA genotypes; 61.75 & 62.35 for POI and 38.3% & 37.7% for control. Furthermore, the rs244715 genotyping showed 172 (57.3%) for TT, 110 (36.3%) for TA, and 18 (6%) for AA in total population. The tousled like kinase 1 (TLK1) gene rs10183486 were significantly associated with POI so that, 33.6% CC, 40.4% CT and 62.5% TT for POI group and 66.4% CC, 59.6% CT and 37.5% TT for control. Allele T and C frequencies for the TMEM rs4806660 were estimated as 65.3% and 34.6%, respectively for the POI group and 73.7% and 26.2%, respectively, for the control group.

**Table 3:** Genotype frequencies in all study participants

Polymorphism Genotype	Total	Genotype frequency in POI, N (%) (Observed)	Genotype Frequency in Control, N (%) (Observed)	*P*-Value	Genotype frequency in POI, N (%) (Expected)	Genotype Frequency in Control, N (%) (Expected)	
rs4806660							
TT	144	50 (34.7)	94 (65.3)	0.020	49.88 (42.64)	99.38 (54.31)	0.2
TC	135	53 (39.3)	82 (60.7)		52.86 (45.18)	70.65 (38.61)	
CC	21	14 (66.7)	7 (33.3)		14.00 (11.97)	12.55 (6.86)	
T		153 (65.3)	270 (73.7)				
C		81 (34.6)	96 (26.2)				
rs451417							
CC	135	58 (35.6)	87 (64.4)	0.001	56.01 (47.88)	85.35 (46.64)	0.1
CA	112	46 (32.1)	76 (67.9)		49.70 (42.48)	78.98 (43.16)	
AA	53	13 (62.3)	20 (37.7)		11.02 (9.42)	18.26 (9.98)	
C		162 (69.2)	250 (68.3)				
A		72 (30.7)	116 (31.6)				
rs16991615							
GG	134	47 (35.8)	96 (64.2)	0.002	38.80 (33.17)	93.01 (50.83)	0.2
GA	119	41 (33.6)	69 (66.4)		57.00 (48.72)	74.62 (40.78)	
AA	47	29 (61.7)	18 (38.3)		20.93 (17.89)	14.95 (8.17)	
G		135 (57.6)	261 (71.3)				
A		99 (42.3)	105 (28.6)				
rs7246479							
TT	125	36 (28.8)	89 (71.2)	0.002	41.27 (35.28)	95.12 (51.98)	0.2
TA	153	67 (43.7)	86 (56.2)		56.28 (48.11)	73.34 (40.08)	
AA	22	14 (63.6)	8 (36.3)		19.18 (16.40)	24.10 (13.17)	
T		139 (59.4)	264 (72.1)				
A		95 (40.5)	102 (27.8)				
rs244715							
AA	172	59 (32.6)	116 (67.4)	0.006	57.33 (49.0)	117.12 (64.0)	0.3
AG	110	46 (44.5)	61 (55.5)		48.97 (41.86)	58.26 (31.84)	
GG	18	12 (66.7)	6 (33.6)		10.45 (8.94)	7.24 (3.96)	
A		164 (70.0)	293 (80.0)				
G		70 (29.9)	73 (19.9)				
rs1046089							
GG	108	37 (34.3)	81 (65.7)	0.200	37.74 (32.26)	85.86 (46.92)	0.1
GA	158	59 (43.7)	89 (56.3)		57.28 (48.96)	78.70 (43.01)	
AA	34	21 (32.4)	13 (67.6)		21.72 (18.57)	19.58 (10.70)	
G		133 (56.8)	251 (68.5)				
A		101 (43.1)	115 (31.4)				
rs2303369							
CC	119	42 (35.3)	77 (64.7)	0.050	40.58 (34.69)	81.16 (44.35)	0.1
CT	144	54 (37.5)	90 (62.5)		56.49 (48.29)	81.16 (44.35)	
TT	37	21 (56.8)	16 (43.2)		19.66 (16.81)	20.27 (11.08)	
C		138 (58.9)	244 (66.6)				
T		96 (41.0)	122 (33.3)				
rs10183486							
CC	140	47 (33.6)	93 (66.4)	0.020	27.22 (23.27)	97.24 (53.14)	0.2
CT	136	55 (40.4)	81 (59.6)		54.01 (46.17)	72.02 (39.36)	
TT	24	15 (62.5)	9 (37.5)		15.28 (13.06)	13.34 (7.29)	
C		149 (63.6)	267 (72.9)				
T		85 (36.3)	99 (27.0)				

POI: premature ovarian insufficiency; Chi-square test has been used

### Association analysis between genotypes and serum vitamin D, calcium, copper, magnesium, zinc, phosphate levels in patient with POI and control

Serum mineral and vitamin D of individuals based on the presence or absence of POI across the genotypes are illustrated in Tables 4 and 5, respectively. Analysis showed that there was significantly difference between calcium level of serum and TMEM variants rs4806660 in participants with POI, although this did not attain significance in control. Conversely, significant difference was shown between the same variant and copper serum level in control. In rs7246479, significant variance with copper level was detected by comparing A/A to T/A and A/A to T/T in control group, whereas for the rs244715 SNP there were significant differences were between the G/G compared with the A/G and the G/G compared to the A/G individuals in the POI group. There was a significant difference between serum phosphate level and MCM8 variants rs451417 only in the patients’ group (CC = 4.3 ± 0.06, CA = 4.2 ± 0.1, AA = 4.7 ± 0.1, *p* = 0.01). Also, there was significant difference between copper serum level and PRRC2A & Fibronectin Type III Domain Containing 4 (FNDC4) variants rs1046089 & rs2303369 respectively, only in the control’ group (*p* = 0.01 for both). No significant differences were found between studied minerals and vitamin D and MCM8 & TLK1 gene variants rs16991615 & rs10183486, respectively.

**Table 4:** Association of genotypes with serum mineral concentrations in the patients with POI (N = 117)

Polymorphism	Zinc	*P*-value	copper	*P*-value	Phosphate	*P*-value	Calcium	*P*-value	Vitamin D	*P*-value	Magnesium	*P*-value
**rs4806660**												
TT	87.37 ± 3.8	NS	104.43 ± 8.5	NS	4.3 ± 0.06	NS	9.6 ± 0.1^b^	**0.002**	22.24 ± 5.2	NS	1.03 ± 0.03	NS
TC	84.26 ± 2.1		107.8 ± 5.3		4.5 ± 014		8.9 ± 0.5^a^		17.58 ± 3.3		1.07 ± 0.03	
CC	87.6 ± 7.6		126.1 ± 10.8		4.6 ± 0.4		10.3 ± 0.2^ab^		15.48 ± 2.5		1.05 ± 0.02	
**rs451417**												
CC	87.7 ± 3.03	NS	108.4 ± 7.3	NS	4.3 ± 0.06^b^	**0.010**	9.5 ± 0.1	NS	21.11 ± 4.4	NS	1.02 ± 0.03	NS
CA	80.9 ± 2.4		116.9 ± 5.8		4.2 ± 0.1^a^		8.2 ± 1.1		15.87 ± 4.8		1.07 ± 0.05	
AA	84.6 ± 4.6		97.3 ± 8.9		4.7 ± 0.1^ab^		9.7 ± 0.1		18.37 ± 4.1		1.08 ± 0.02	
**rs16991615**												
GG	87.3 ± 3.07	NS	103.3 ± 7.07	NS	4.5 ± 0.12	NS	9.6 ± 0.1	NS	23.2 ± 4.4	NS	1.07 ± 0.03	NS
GA	86.04 ± 3.3		11.3 ± 6.4		4.4 ± 1.3		9.4 ± 0.2		13.7 ± 4.8		1.03 ± 0.04	
AA	87.26 ± 3.2		113.5 ± 9.4		4.8 ± 0.17		8.6 ± 1.08		17.35 ± 4.1		1.03 ± 0.03	
**rs7246479**												
TT	87.3 ± 3.8	NS	104.4 ± 8.5	NS	4.3 ± 0.06	NS	9.6 ± 0.1	NS	22.2 ± 5.2	NS	1.04 ± 0.06	NS
TA	84.2 ± 2.1		107.8 ± 5.3		4.5 ± 0.1		8.9 ± 0.5		17.5 ± 3.3		1.03 ± 0.03	
AA	78.6 ± 7.6		126.1 ± 10.8		4.6 ± 0.4		10.3 ± 0.2		15.4 ± 2.5		1.07 ± 0.03	
**rs244715**												
AA	87.4 ± 2.7	NS	99.4 ± 6^b^	**0.007**	4.5 ± 0.1	NS	9.4 ± 0.1	NS	21.9 ± 5	NS	1.07 ± 0.03	NS
AG	84.9 ± 3.1		108.9 ± 4.6^a^		4.4 ± 0.1		8.8 ± 0.7		16.8 ± 3.3		1.04 ± 0.02	
GG	73.5 ± 3		142 ± 10.3^ab^		4.4 ± 0.2		10.3 ± 0.2		17.04 ± 2.1		0.9 ± 0.04	
**rs1046089**												
GG	94.8 ± 3.1	NS	103.4 ± 7.6	NS	4.4 ± 0.09	NS	9.5 ± 0.1	NS	25.4 ± 4.8	NS	1.04 ± 0.02	NS
GA	85.9 ± 2.7		110.3 ± 5.5		4.5 ± 0.1		8.9 ± 0.6		15.6 ± 3		1.07 ± 0.04	
AA	77.4 ± 3.5		117.1 ± 14.2		4.3 ± 0.1		10.0 ± 0.5		11.4 ± 5.5		1.05 ± 0.02	
**rs2303369**												
CC	84.8 ± 2.8	NS	114.2 ± 7.2	NS	4.1 ± 0.1	NS	9.3 ± 0.2	NS	20.1 ± 5.6	NS	0.9 ± 0.04	NS
CT	84.8 ± 3.1		106.8 ± 6.3		4.5 ± 0.1		9.6 ± 0.09		18.9 ± 3.3		1.09 ± 0.02	
TT	83.6 ± 3.3		102.07 ± 9.07		4.6 ± 0.1		8.2 ± 1.7		17.06 ± 5.9		1.03 ± 0.3	
**rs10183486**												
CC	82.3 ± 2.4	NS	106.7 ± 6.4	NS	4.3 ± 0.09	NS	9.03 ± 0.54	NS	20.9 ± 3.5	NS	1.04 ± 0.03	NS
CT	87.3 ± 3.09		112.2 ± 6.5		4.4 ± 0.12		9.5 ± 0.18		17.39 ± 4.2		1.03 ± 0.03	
TT	83.5 ± 7.2		102.3 ± 12.4		5.0 ± 0.3		9.7 ± 0.35		18.4 ± 2.9		1.14 ± 0.03	

One-way Analysis of Variance (ANOVA) and Tukey tests were applied; Data are represented as Mean ± SD (Standard Deviation); All data regarding the concentrations of all micronutrients had normal distribution. a,b & c:same letters show significant differences between genotypes

**Table 5:** Association of genotypes with serum minerals in the control participants (N = 183)

Polymorphism	Zinc	*P*-value	Copper	*P*-value	Phosphate	*P*-value	Calcium	*P*-value	Vitamin D	*P*-value	Magnesium	*P*-value
**rs4806660**												
TT	82.63 ± 1.7	NS	98.7 ± 3.6^a^	**0.010**	4.5 ± 0.2	NS	9.5 ± 0.3	NS	12.85 ± 2.5	NS	1.08 ± 0.07	NS
TC	84.82 ± 1.8		112.19 ± 3.5^b^		4.7 ± 0.2		9.7 ± 0.1		9.48 ± 2.3		1.08 ± 0.03	
CC	88.23 ± 3.6		127.6 ± 23.8^ab^		4.5 ± 0.2		9.3 ± 0.2		10.45 ± 2.1		1.07 ± 0.03	
**rs451417**												
CC	84.4 ± 1.9	NS	110.4 ± 3.3	NS	4.8 ± 0.1	NS	9.6 ± 0.2	NS	12.6 ± 3.1	NS	1.07 ± 0.03	NS
CA	83.7 ± 1.8		104.9 ± 4.6		4.4 ± 0.3		9.9 ± 0.6		10.2 ± 1.7		1.09 ± 0.09	
AA	81.8 ± 3.2		93.5 ± 7.9		4.4 ± 0.5		8.7 ± 0.7		11.2 ± 4.7		1.06 ± 0.06	
**rs16991615**												
GG	83.9 ± 1.9	NS	102.06 ± 4.1	NS	4.69 ± 0.23	NS	9.5 ± 0.2	NS	23.2 ± 4.4	NS	1.09 ± 0.03	NS
GA	83.8 ± 1.6		109.9 ± 3.6		4.61 ± 0.34		9.8 ± 0.5		13.7 ± 4.8		1.06 ± 0.08	
AA	83.9 ± 4.5		109.2 ± 9.6		4.67 ± 0.21		9.6 ± 0.3		17.35 ± 4.1		1.13 ± 0.03	
**rs7246479**												
TT	82.6 ± 1.8	NS	98.7 ± 3.6^a^	**0.010**	4.3 ± 0.3	NS	9.6 ± 0.2	NS	10.2 ± 2.2	NS	1.08 ± 0.03	NS
TA	84.8 ± 1.8		112.1 ± 3.5		4.5 ± 0.2		9.5 ± 0.4		12.8 ± 2.5		1.08 ± 0.07	
AA	88.2 ± 3.6		127.6 ± 23.8^a^		4.7 ± 0.2		9.7 ± 0.1		9.4 ± 2.3		1.02 ± 0.04	
**rs244715**												
AA	84.1 ± 1.5	NS	102.8 ± 3.1	NS	4.5 ± 0.3	NS	9.5 ± 0.4	NS	10.6 ± 1.7	NS	1.09 ± 0.8	NS
AG	84.4 ± 2.3		112.5 ± 4.9		4.9 ± 0.2		9.7 ± 0.2		14.3 ± 4.7		1.07 ± 0.03	
GG	74.4 ± 1.5		118.5 ± 14.4		4.4 ± 0.3		9.6 ± 0.2		12.2 ± 3.6		1.06 ± 0.07	
**rs1046089**												
GG	82.5 ± 1.7	NS	103.3 ± 3.9^b^	**0.010**	4.3 ± 0.2	NS	9.5 ± 0.4	NS	12.2 ± 2.3	NS	1.05 ± 0.2	NS
GA	83.8 ± 1.9		103.9 ± 3.6^a^		4.3 ± 0.4		9,6 ± 0.8		13.5 ± 2.8		1.1 ± 0.1	
AA	89.4 ± 3.2		128 ± 8.4^ab^		4.7 ± 0.1		9.6 ± 0.1		10.7 ± 2.6		1.07 ± 0.03	
**rs2303369**												
CC	82.9 ± 1.7	NS	105.5 ± 3.9^b^	**0.010**	4.2 ± 0.4	NS	9.6 ± 0.7	NS	12.2 ± 2.7	NS	1.2 ± 0.1	NS
CT	84.1 ± 1.9		102.8 ± 3.7^a^		4.3 ± 0.4		9.5 ± 0.8		13.5 ± 2.8		1.1 ± 0.1	
TT	87.7 ± 2.6		135.1 ± 9.2^ab^		4.7 ± 0.1		9.6 ± 0.1		10.7 ± 2.6		1.07 ± 0.03	
**rs10183486**												
CC	82.8 ± 1.8	NS	109.1 ± 4.3	NS	4.7 ± 0.23	NS	9.6 ± 0.19	NS	10.4 ± 2.3	NS	1.08 ± 0.03	NS
CT	84.1 ± 1.7		103.2 ± 3.4		4.6 ± 0.2		9.6 ± 0.47		12.6 ± 2.8		1.08 ± 0.04	
TT	90.3 ± 5.01		106.1 ± 1.6		4.5 ± 0.23		9.5 ± 0.3		12.2 ± 2.2		1.07 ± 0.07	

One-way Analysis of Variance (ANOVA) and Tukey tests were applied; Data are represented as Mean ± SD (Standard Deviation); All data regarding the concentrations of all micronutrients had normal distribution. a,b & c:same letters show significant differences between genotypes

### The significant association between SNPs associated with POI and copper

Multinomial regression analysis was used to predict the association of 5 variants with serum Cu (µg/dL) in patient and control groups (Table 6). We categorized serum copper levels into three categories; low < 80 µg/dL, moderate 80–155 µg/dL, high > 155 µg/dL. The adjusted model included traditional risk factors for POI. We found that participants with POI who carried the C allele of rs4806660 variant had lower serum copper than those with TT genotypes (OR = 0.22 (0.11–0.81), *p* = 0.03), although this did not attain significance in individual without POI (*p* = 0.3). According to analysis of the BRSK1 gene polymorphism, rs7246479, healthy individuals with A/A genotype are more likely to have high copper level (OR = 41.08 (3.7-450.8), *p* = 0.002) than patients with T/T genotype. This indicates that the A allele could be a promoting allele for a high serum copper in healthy people. Whilst, the rs244715 polymorphism, patients with the G/G genotype were more likely to have higher serum copper levels (OR = 6.7 (1.3–24.3), *p* = 0.02) than patients with the A/A genotype. This means that the effect of G allele on serum copper level may be in the homozygous state among patient with POI. After regression analysis, two SNPs (rs1046089, rs2303369) were not in significant associations with serum copper concentration among person with and without POI (Table 6).

**Table 6:** Multivariable logistic regression analysis; Most common genotypes significantly associated with the minerals

Polymorphism (Reference Dominant Homozygous genotype)	Serum (µg/dL)	POI cases				Control			
		OR* (95% CI)	*P*	OR* (95% CI)	*P*	OR* (95% CI)	*P*	OR* (95% CI)	*P*
**rs4806660 (TT)**	**Cu**	**TC**		**CC**		**TC**		**CC**	
	˂80	0.22 (0.11–0.81)	**0.03**	0.78 (0.12–2.6)	0.6	0.6 (0.25–1.6)	0.3	3.2 (0.4–24)	0.2
	80–155	Ref		Ref		Ref		Ref	
	> 155	0.17 (0.01–1.5)	0.1	0.32 (0.03–3.02)	0.3	1.2 (0.11–13.01)	0.8	0.5 (0.16–1.8)	0.3
**rs7246479 (TT)**	**Cu**	**TA**		**AA**		**TA**		**AA**	
	˂80	1.4 (0.49–4.5)	0.4	0.38 (0.03–3.7)	0.4	0.8 (0.3-2)	0.6	2.7 (0.2–34.1)	0.4
	80–155	Ref		Ref		Ref		Ref	
	> 155	1.2 (0.3–5.05)	0.7	0.9 (0.1–7.2)	0.9	3.04 (0.7–12.1)	0.1	41.08 (3.7-450.8)	**0.002**
**rs244715 (AA)**	**Cu**	**AG**		**GG**		**AG**		**GG**	
	˂80	0.36 (0.12–1.05)	0.06	0.29 (0.03–2.7)	0.2	1.7 (0.3–3.6)	0.9	5.7 (0.7-45.06)	0.09
	80–155	Ref		Ref		Ref		Ref	
	> 155	1.2 (0.28–5.3)	0.7	6.7 (1.3–24.3)	**0.02**	0.6 (0.2–1.8)	0.4	1.17 (0.11–12.3)	0.8
**rs1046089 (GG)**	**Cu**	**GA**		**AA**		**GA**		**AA**	
	˂80	0.7 (0.25–2.05)	0.5	1.19 (0.2–6.4)	0.8	0.7 (0.3–1.9)	0.5	0.7 (0.13–3.6)	0.6
	80–155	Ref		Ref		Ref		Ref	
	> 155	0.6 (0.1–2.3)	0.5	1.2 (0.17–8.8)	0.8	0.3 (0.08–1.2)	0.9	1.8 (0.4–8.1)	0.3
**rs2303369 (CC)**	**Cu**	**CT**		**TT**		**CT**		**TT**	
	˂80	0.4 (0.1–1.5)	0.2	0.2 (0.04–1.2)	0.08	0.8 (0.3–2.1)	0.7	0.5 (0.06–5.06)	0.6
	80–155	Ref		Ref		Ref		Ref	
	> 155	0.5 (0.1–2.1)	0.4	0.2 (0.02–2.03)	0.1	0.2 (0.07–1.11)	0.07	2.01 (0.3–10.7)	0.4
**rs4806660 (TT)**	**Calcium**	**TC**		**CC**		**TC**		**CC**	
	9.5>	13.7 (0.2–65.6)	0.1	0.07 (0.002-3.4)	0.1	7.8 (0.98–34.3)	0.9	6.8 (0.64–72.8)	0.1
	9.5<	Ref		Ref		Ref		Ref	
**rs451417 (AA)**	**Phosphate**	**CC**		**CA**		**CC**		**CA**	
	4.5>	10.5 (0.2–45.7)	0.2	3.2 (0.09–10.8)	0.5	2.02 (0.1–39.3)	0.6	0.4 (0.04–4.2)	0.4
	4.5<	Ref		Ref		Ref		Ref	

POI: Premature Ovarian Insufficiency, OR: Odds Ratio, CI: Confidence Interval; Multivariable logistic regression analysis was performed after adjusting for confounding factors including age and BMI; Dominant homozygous of each SNPs was considered as the reference genotype in the regression analysis

## Discussion

In this study we selected several SNPs that have previously been shown to be associated with POI, including: rs16991615, rs244715, rs451417, rs7246479, rs4806660, rs10183486, rs2303369 and rs1046089. All of these SNPs were found to be associated with POI apart from the rs2303369 and rs1046089 polymorphisms and POI. These SNPs are located on different loci in the genome for example, rs1046089, rs2303369, rs10183486 and rs244715 are located on PRRC2A, Fibronectin Type III Domain Containing 4 (FNDC4), TLK1 and Zinc Finger Protein 346 (ZNF346), respectively while rs16991615 and rs451417 are located on MCM8. In addition to rs7246479 and rs4806660 are both located on TMEM150B. It was also similar to GWAS population due to the proximity of MAF of us to the GWAS. We investigated the genotype frequency for the POI and the control groups, and evaluated the association of genotypes with serum minerals in the two groups. To our knowledge, for the first time in this study, we have investigated the relationship between minerals with vitamin D and variants associated with POI.

Various studies have investigated the role of genetic variants in the occurrence of POI. Approximately 30–85% of the onset menopausal age is related to heredity, and remarkable amount (15–30%) of POF cases are familial [46]. The rs4806660 is located on chromosome 19 containing TMEM150B, which encodes the transmembrane protein 150B, also known damage-regulated autophagy modulator 3 (DRAM3). This protein is involved in autophagy and apoptosis [47, 48]. Expression of TMEM150B leads to accumulation of autophagosomes in basal conditions and increases the autophagy flux, while removal of TMEM150B interrupts the autophagy flux and confirms that it modulates macro autophagy [47]. Our finding indicated that there was significantly difference between serum calcium and TMEM variants rs4806660 in participants with POI, although this did not attain significance in control. Also, there was no significant difference between serum minerals and vitamin D and MCM8 & TLK1 gene variants rs16991615 and rs10183486, respectively. Calcium and vitamin D have been implicated in several obstetric diseases, including polycystic ovary syndrome, endometriosis, and premenstrual syndrome, and appear to be involved in fertility [49]. Carwile et al. [50] did not find any relationship between calcium intake and age at menopause. Purdue-Smithe et al. found that high doses calcium intake and vitamin D were associated with lower risk of early menopause and they may play a role in reducing the risk of early menopause [49]. Also analysis showed a significant difference between phosphate serum level and MCM8 variants rs451417 in the POI group. MCM8 is located on chromosome 19 that coding DNA replication licensing factor MCM8 and acts in DNA repair and also MCM8 play role in gametogenesis [51, 52]. Although Kebapcillar et al. showed that serum zinc was significantly lower in POI cases than controls [53], we found no significant relationship between serum zinc and the POI-related genetic variants.

Another SNP is rs7246479 which is located in BRSK1, a gene is most commonly expressed in the brain and to a lesser extent in the ovaries and BRSK1 is thought to stimulate gonadotropin-releasing hormone secretion, leading to POI [47]. We observed significant variance with copper level was detected by comparing A/A to T/A and A/A to T/T in control group, whiles in rs244715 significant differences between copper with G/G compared with A/G and G/G compared to A/G only in POI group. The rs244715 is located in ZNF346 that encodes a double-stranded RNA binding protein Which is probably involved in apoptosis regulation [54, 55]. Furthermore, we observed a significant difference between copper serum level and PRRC2A & FNDC4 variants rs1046089 & rs2303369 respectively, only in the control group.

We also examined the association between SNPs associated with POI and copper, calcium, phosphate including rs4806660, rs7246479, rs244715, rs1046089, rs2303369, and rs451417. We found that the C allele of rs4806660 variant was associated with lower serum copper values than those with TT genotypes in the POI group, while this did not achieve significance in the control group. In rs7246479 that is located in BRSK1 gene, participants who have a normal menopause with A/A genotype are more likely to have high copper level than patients with T/T genotype. This suggests that the A allele may be involved in raising serum copper in healthy individuals. Besides, in rs244715 the analysis indicated that in the POI group with the G/G genotype have higher serum copper than patients with the A/A genotype. This implies the effect of G allele on copper level must be in the homozygous condition in POI group in two SNPs including rs1046089, rs2303369, we did not find significant associations with serum copper among POI or the control group. Also, in regression analysis we did not find any associations between serum calcium and phosphate and the SNPs (rs4806660, rs451417).

A possible explanation for this might be that these SNPs, which are associated with POI, cause hormonal changes that leads to altered metabolism [1, 56]. Menopausal changes in trace mineral status may affect the pathology of premature menopausal diseases [56]. Bednarek-Tupikowska et al. conducted a study on women during sex hormone therapy that measured the level of different minerals in pre- and postmenopausal women. They showed that estrogen administration in postmenopausal women tended to increase serum copper levels [56]. Berg et al. found that oral contraceptive administration is associated with increased serum copper levels [57]. However, in another study, no significant relationship was observed between estrogen administration and copper levels [58]. In another study, serum copper levels were significantly associated with reproductive health problems in women [59]. Women with POF had significantly higher copper levels than a control group [24]. Ferdous et al. stated that serum copper levels were significantly higher in postmenopausal women than in the premenopausal [60]. In contrast, Mutlu et al. did not find any significant difference between copper levels in pre- and postmenopausal women [61].

Copper plays an important role in various metabolic functions [59]. It is also involved in the development and maintenance of the immune system [24]. The relationship between copper and menopause was also expressed in studies. These results suggest that, in general, hormonal changes may be related to serum copper levels. Therefore, some variants associated with POI and POI associated with hormonal changes [62]. Hormonal changes can be linked to variation of micronutrient levels. However, further studies are needed on the association of serum levels of various minerals with POI.

There are some limitations to this study. First, it was a cross sectional study that includes groups of case and control and therefore we cannot attribute causality. Second, we did not consider the other SNPs that is possible associated with POI. Third dietary mineral intake was not considered, which could affect the results. We recommend future researches conduct longitudinal studies such as case-control or cohort with larger sample size and consider other associated SNPs. Also they can investigate the effect of nutritional interventions on these variants and POI in randomized clinical trials.

In conclusion, serum calcium, phosphate and copper in POI patients were significantly different in women with different rs4806660, rs451417, rs244715, SNPs respectively. Also, for the rs4806660, rs7246479, rs1046089, rs2303369, SNPs there was a significant difference between serum copper and SNPs in the control group. Regression analysis showed that the patients with C allele of rs4806660 had lower serum copper than patients with T/T genotype. Furthermore, the healthy participants with A/A genotype of rs7246479 and POI individuals with the G/G genotype of rs244715 probably have higher copper than individuals with the T/T genotype and the A/A genotype, respectively. This study suggests a potential mechanism in the pathogenesis of gene-related cases of POI. Further studies of the mechanisms involved in POI will require longitudinal studies.

### Electronic supplementary material

Below is the link to the electronic supplementary material.

Supplementary Material 1: Association of genotypes with serum mineral concentrations in total population

## Data Availability

The datasets generated during the current study are available from the corresponding author upon reasonable request. Data sharing is not applicable to this article as no datasets were generated or analyzed during the current study.
