# Supplementary material for: Micronutrients intake and genetic variants associated with premature ovarian insufficiency; MASHAD cohort study
Source: BMC Womens Health. 2024 Feb 4;24:91. doi: 10.1186/s12905-023-02865-4 (PMC10840145; doi:10.1186/s12905-023-02865-4)
Supplement: Supplementary file 1 — Supplementary Material 1: Association of genotypes with serum mineral concentrations in total population [file 12905_2023_2865_MOESM1_ESM.docx]

| **Supplementary 1.** *Association of genotypes with serum mineral concentrations in total population* | | | | | | | | | | | | |
| --- | --- | --- | --- | --- | --- | --- | --- | --- | --- | --- | --- | --- |
| **Polymorphism** | **Zinc** | P-value | **copper** | P-value | **Phosphate** | P-value | **Calcium** | P-value | **Vitamin D** | P-value | **Magnesium** | P-value |
| **rs4806660** | | | | | | | | | | | | |
| TT | 84.13±15.69 | NS | 104.22 ±36.1 | NS | 4.32 ±0.8 | NS | 9.43 ±1.32 | **0.004** | 15.18±12.95 | NS | 1.06 ±0.21 | NS |
| TC | 84.28±18 |  | 109.38±36.36 |  | 4.68±0.55 |  | 9.72±0.54 |  | 16.25±11.59 |  | 1.08±0.13 |  |
| CC | 83.81±15.6 |  | 116.27±43.35 |  | 4.8±0.17 |  | 6.83±4.98 |  | 15 ±4.24 |  | 1.06 |  |
| **rs451417** | | | | | | | | | | | | |
| CC | 85.58±17.63 | NS | 109.77±36.03 | NS | 4.61 ±0.55 | NS | 9.60±0.66 | NS | 16.90±13.78 | NS | 1.05±0.14 | NS |
| CA | 82.8±14.35 |  | 109.14±36.07 |  | 4.38±0.85 |  | 9.16±2.59 |  | 12.83±8.79 |  | 1.09±0.24 |  |
| AA | 83.39±18.95 |  | 95.66±38.54 |  | 4.69±0.56 |  | 9.49±0.83 |  | 16.8±10.36 |  | 1.08±0.08 |  |
| **rs16991615** | | | | | | | | | | | | |
| GG | 85.06±17.40 | NS | 102.5±37.50 | NS | 4.59 ±0.64 | NS | 9.55 ±0.74 | NS | 18.24±13.72 | NS | 1.08±0.12 | NS |
| GA | 84.58±15.76 |  | 110.43±33.07 |  | 4.53±0.85 |  | 9.67±1.36 |  | 12.78±8.76 |  | 1.05±0.23 |  |
| AA | 80.70±16.97 |  | 111.7±42.41 |  | 4.54±0.46 |  | 8.96±2.52 |  | 14.89±11.53 |  | 1.06±0.09 |  |
| **rs7246479** | | | | | | | | | | | | |
| TT | 83.97±17.35 | NS | 100.39±36.34 | **0.01** | 4.47 ±0.63 | NS | 9.6 ±1.24 | NS | 16.53±12.98 | NS | 1.06 ±0.20 | NS |
| TA | 84.58±15.78 |  | 110.34±35.13 |  | 4.63±0.70 |  | 9.27±1.69 |  | 14.88±11.31 |  | 1.08±0.12 |  |
| AA | 82.24 ±20.06 |  | 126.69±42.83 |  | 4.6±0.56 |  | 10.3±0.28 |  | 15.48±3.56 |  | 1.03 |  |
| **rs244715** | | | | | | | | | | | | |
| AA | 85.14±16.55 | **0.023** | 101.80±34.81 | **0.001** | 4.52±0.77 | NS | 9.53±1.21 | NS | 15.84±13.20 | NS | 1.09±0.21 | NS |
| AG | 84.67±17.56 |  | 110.85±37.28 |  | 4.64±0.58 |  | 9.26±1.90 |  | 15.86±11.36 |  | 1.06±0.10 |  |
| GG | 73.84±8.61 |  | 134.17±36.42 |  | 4.43±0.48 |  | 9.82±0.49 |  | 14.54±5.84 |  | 1.04±0.13 |  |
| **rs1046089** | | | | | | | | | | | | |
| GG | 83.28±15.57 | NS | 103.40 ±36.04 | **0.034** | 4.41±0.77 | NS | 9.58±1.30 | NS | 20.54±13.73 | NS | 1.07±0.21 | NS |
| GA | 84.69±18.11 |  | 106.61±35.55 |  | 4.67±0.59 |  | 9.34±1.65 |  | 13.12±10.16 |  | 1.07±0.14 |  |
| AA | 85.02±13.66 |  | 123.96±40.85 |  | 4.43±0.25 |  | 9.77±0.64 |  | 11.60±7.77 |  | 1.05±0.02 |  |
| **rs2303369** | | | | | | | | | | | | |
| CC | 83.58±15.41 | NS | 108.46±36.03 | NS | 4.26±0.89 | NS | 9.47 ±1.47 | NS | 17.25±13.61 | NS | 1.05±0.24 | NS |
| CT | 84.42±18.61 |  | 104.37±37.14 |  | 4.69±0.54 |  | 9.64±0.55 |  | 14.69±11.22 |  | 1.08±0.12 |  |
| TT | 85.40±11.43 |  | 116.08±36.66 |  | 4.58±0.28 |  | 8.42±3.61 |  | 16.05±10.51 |  | 1.04±0.05 |  |
| **rs10183486** | | | | | | | | | | | | |
| CC | 82.67±16.05 | NS | 108.30±39.78 | NS | 4.56±0.65 | NS | 9.29±1.68 | NS | 16.99±12.67 | NS | 1.07 ±0.13 | NS |
| CT | 85.35±16.87 |  | 106.61±34.47 |  | 4.50±0.67 |  | 9.63±1.28 |  | 14.70±11.14 |  | 1.06±0.20 |  |
| TT | 86.75±19.38 |  | 104±28.86 |  | 5.0±0.62 |  | 9.7±0.61 |  | 7.19 |  | 1.15±0.07 |  |
